# Meta-analysis of molecular imaging of translocator protein in major depression

**DOI:** 10.3389/fnmol.2022.981442

**Published:** 2022-09-26

**Authors:** Benjamin Eggerstorfer, Jong-Hoon Kim, Paul Cumming, Rupert Lanzenberger, Gregor Gryglewski

**Affiliations:** ^1^Department of Psychiatry and Psychotherapy, Comprehensive Center for Clinical Neurosciences and Mental Health (C3NMH), Medical University of Vienna, Vienna, Austria; ^2^Department of Psychiatry, Gachon University College of Medicine, Gil Medical Center, Neuroscience Research Institute, GAIHST, Gachon University, Incheon, South Korea; ^3^Department of Nuclear Medicine, Inselspital, Bern University, Bern, Switzerland; ^4^School of Psychology and Counselling, Queensland University of Technology, Brisbane, QLD, Australia

**Keywords:** depression, meta-analysis, molecular imaging, neuroinflammation, positron emission tomography, translocator protein

## Abstract

Molecular neuroimaging studies provide mounting evidence that neuroinflammation plays a contributory role in the pathogenesis of major depressive disorder (MDD). This has been the focus of a number of positron emission tomography (PET) studies of the 17-kDa translocator protein (TSPO), which is expressed by microglia and serves as a marker of neuroinflammation. In this meta-analysis, we compiled and analyzed all available molecular imaging studies comparing cerebral TSPO binding in MDD patients with healthy controls. Our systematic literature search yielded eight PET studies encompassing 238 MDD patients and 164 healthy subjects. The meta-analysis revealed relatively increased TSPO binding in several cortical regions (anterior cingulate cortex: Hedges’ *g* = 0.6, 95% CI: 0.36, 0.84; hippocampus: *g* = 0.54, 95% CI: 0.26, 0.81; insula: *g* = 0.43, 95% CI: 0.17, 0.69; prefrontal cortex: *g* = 0.36, 95% CI: 0.14, 0.59; temporal cortex: *g* = 0.39, 95% CI: –0.04, 0.81). While the high range of effect size in the temporal cortex might reflect group-differences in body mass index (BMI), exploratory analyses failed to reveal any relationship between elevated TSPO availability in the other four brain regions and depression severity, age, BMI, radioligand, or the binding endpoint used, or with treatment status at the time of scanning. Taken together, this meta-analysis indicates a widespread ∼18% increase of TSPO availability in the brain of MDD patients, with effect sizes comparable to those in earlier molecular imaging studies of serotonin transporter availability and monoamine oxidase A binding.

## Introduction

A constellation of molecular, inflammatory, and metabolic alterations is widely held to be relevant in the pathophysiology of major depressive disorder (MDD) (Otte et al., 2016), an often-devastating psychiatric condition with a world-wide prevalence of about 5% (Ferrari et al., 2013). The composite of these alterations may manifest in perturbation of normal neurotransmission and neuroplasticity, associated with morphological and functional changes in multiple structures and functional networks of the brain, as shown by neuroimaging studies (Schmaal et al., 2020). Compelling evidence derived from genome-wide association studies, epidemiological studies, and randomized controlled trials suggests involvement of the immune system in a variety of psychiatric disorders, including psychotic disorders and mood disorders such as MDD (The Network, and Pathway Analysis Subgroup of the Psychiatric Genomics Consortium, 2015; Hughes and Ashwood, 2020). An involvement of the immune system is implied by the broad similarity between core symptoms of MDD and so-called “sickness behavior” that occurs during an acute inflammatory state (Dantzer et al., 2008). Furthermore, a comprehensive review of laboratory findings in patients with MDD showed elevated peripheral proinflammatory markers such as C-reactive protein (CRP), certain interleukins, and tumor necrosis factor alpha (TNF-α), all of which is consistent with the occurrence of a pro-inflammatory state (Osimo et al., 2020). Support for a neuroinflammatory component of MDD is also provided by *post-mortem* studies showing elevated immune cytokine levels in various brain regions, and likewise by findings of raised inflammatory markers in the cerebrospinal fluid of MDD patients (Enache et al., 2019).

Peripheral inflammation might trigger a central neuroinflammatory reaction via several mechanisms. One candidate mechanism entails a humoral pathway whereby a leaky blood-brain barrier is permissive to the entry of cytokines from the circulation into the central nervous system (CNS) (Huang et al., 2021). Other propose a neural pathway whereby afferent nerves convey peripheral cytokine signals to the CNS (Miller et al., 2009), or communication along the gut-brain axis (Li et al., 2022a). In addition, a central inflammatory process could arise via immunologically active cells in the CNS (Miller and Raison, 2016).

Microglia are the resident macrophages of the brain, accounting for 5–10% of all cells in the central nervous system (Pelvig et al., 2008; Mondelli et al., 2017). In the healthy CNS, microglia are habitually present in a dormant state with ramified morphology, but nonetheless release neurotrophic factors that contribute to the regulation of synaptic homeostasis, especially in the contexts of neurotoxic or traumatic brain injury (Rodríguez-Gómez et al., 2020; Bravo et al., 2022). Indeed, a diverse range of factors can provoke microglial activation and changes in morphological phenotype (Wolf et al., 2017), for example in response to a high dietary intake of sucrose (Patkar et al., 2021), which is a characteristic of modern western diets. Activated microglia promote an inflammatory cascade involving the release of cytokines, chemokines, and other inflammatory mediators such as nitric oxide and reactive oxygen species, which together trigger reciprocal activation of astroglia, thus amplifying CNS inflammatory responses after an initial insult (Miller et al., 2009).

The expression of the 18-kDA translocator protein (TSPO) in the mitochondrial membrane of resting microglia increases as part of neuroinflammatory reactions. Numerous radiotracers have been characterized for single-photon emission computed tomography (SPECT) and positron emission tomography (PET) studies of TSPO, including [^11^C]PK11195, [^18^F]FEPPA and [^11^C]PBR28 (Cumming et al., 2018). Although inferences related to inflammatory states and TSPO should consider that TSPO expression is not unique to microglia and that elevated TSPO binding in humans could also be attributable to local proliferation of myeloid cells or increased recruitment of monocytes (Narayan et al., 2017; Owen et al., 2017), TSPO remains the most widely used marker of inflammation in the living brain in diverse studies of neuropsychiatric disorders, including MDD (Mondelli et al., 2017; Enache et al., 2019).

A first systematic review on this topic appearing in 2021 revealed a relatively small number of TSPO PET studies investigating microglial reactions in patients with MDD (Gritti et al., 2021). Furthermore, published findings were not entirely consistent, and have not hitherto included all relevant TSPO PET studies (Enache et al., 2019; Schubert et al., 2021a). Hence, we aimed in this meta-analysis to analyze all available TSPO PET studies comparing TSPO binding in MDD vs. healthy control groups. We also undertook an exploratory search for possible associations of TSPO PET results with depression severity, body mass index (BMI), and other factors.

## Materials and methods

### Data collection

**FIGURE 1:**
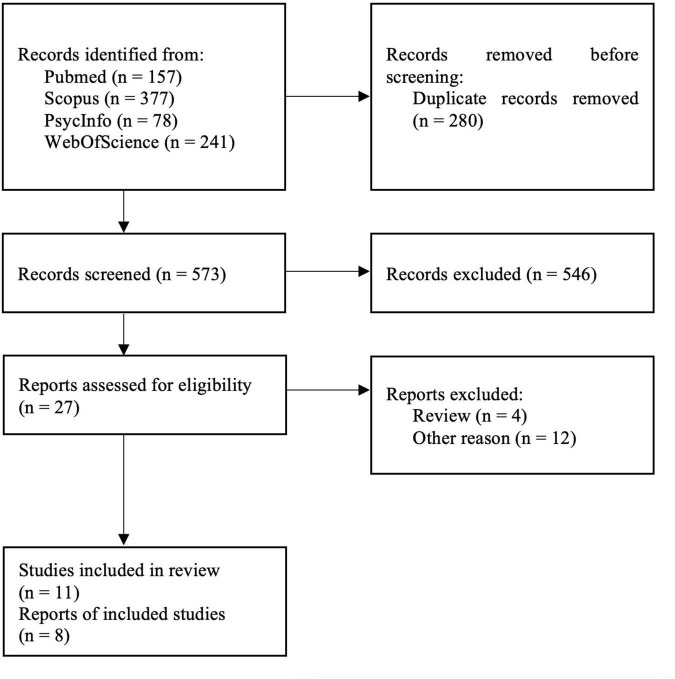
PRISMA flowchart showing the inclusion of studies for the meta-analysis (Moher et al., 2009).

The bibliographic databases PubMed, Scopus, PsycInfo, and Web of Science were searched systematically using the terms (“mood disorders” or “affective disorders” or “depression” or “major depressive disorder” or “bipolar disorder” or “major depressive episode”) and (“neuroinflammation” or “inflammation” or “microglia” or “TSPO” or “translocator protein”) and (“positron emission tomography” or “PET” or “molecular imaging”) in December 2021. The literature search was conducted in accordance with the guidelines of the PRISMA statement (Moher et al., 2009), and the selection of included studies was performed using the Rayyan software (Ouzzani et al., 2016). Studies were included if they fulfilled the following inclusion criteria: peer-reviewed English or German language original articles, *in vivo* TSPO studies, and studies that reported means and standard deviation (SD) values or effect sizes of molecular imaging outcome measures reflecting cerebral TSPO binding in groups of patients with unipolar depression and healthy controls for several different brain regions. Exclusion criteria were case studies, reviews and meta-analyses, pre-clinical studies, *post-mortem* studies, and studies in patients with a diagnosis of comorbid psychiatric, somatic, or neurological disorders. The systematic search as well included SPECT studies, although no such studies emerged. Figure 1 shows the detailed selection of publications in a flow chart based on the PRISMA statement (Moher et al., 2009). In case of overlapping samples, the study with largest sample size was selected. The corresponding author of one eligible publication shared data for additional regions based on the Hammers atlas (Hammers et al., 2003; Su et al., 2016). Results of a study that reported results separately for both hemispheres were averaged across hemispheres (Joo et al., 2021). Results from a study that separately reported results for medically treated and untreated MDD patients in groups of equal size were averaged to a single patient group (Setiawan et al., 2018). For one study that reported results in graphical format only (Hannestad et al., 2013), mean and SD values were estimated using WebPlotDigitizer, version 4.5 (Rohatgi, 2021). Demographic variables (age, sex, BMI), depression severity, type of tracer, type of outcome measure, and psychiatric medication history (medication-free interval, number of medication-free subjects) were extracted from the studies included in our analyses. For studies that reported Montgomery–Åsberg Depression Rating Scale (MADRS) score only (Hannestad et al., 2013; Su et al., 2016; Richards et al., 2018), the equivalent Hamilton Depression Rating Scale (HDRS) score, as provided by the conversion of Leucht et al. (2018), was used for further calculations.

### Statistics

The statistical analysis was conducted using R 4.1.2 and the metafor package, version 3.0-2 (Viechtbauer, 2010).

TSPO PET studies can report several outcome measures, including the binding potential (BP_*ND*_), which describes the specific binding relative to a reference tissue. In cases where the arterial input function has been measured, the preferred endpoint is the equilibrium distribution volume of the tracer in brain (V_*T*_; ml/g), which is sometimes corrected for the plasma-free fraction (V_*T*_/*f*_*P*_). The diversity of outcome measures makes it compulsory to calculate standardized effect size estimates to allow their combination in a meta-analysis. Consequently, we calculated Hedges’ *g* (Hedges, 1981), the standardized mean difference between MDD and control groups for each dataset and selected brain region. Another factor is the polymorphism of TSPO in certain populations, which imparts low specific binding of most second-generation PET TSPO tracers to carriers of the rs6971 allele (Owen et al., 2012). Therefore, in this study we used endpoints that had been corrected for group composition with respect to the high and low binding alleles. To gauge the overall relative increase in TSPO in depressed individuals, the percentage increase in mean TSPO binding was calculated for each study, weighted for sample size and averaged across all reported brain regions.

Meta-analyses were performed separately for each of five brain regions that were reported in at least three published studies, namely anterior cingulate cortex, prefrontal cortex, insula, hippocampus, and temporal cortex. We applied a random effects model with restricted maximum likelihood estimation of variance. In a random-effects model, the weighting of the individual study estimates are inversely proportional to the sum of their sample variance (*f*_*i*_) and the between-study variance (*f*^2^). To gauge variation of study results caused by between-study heterogeneity, we calculated Higgins’ I^2^ (Higgins and Thompson, 2002). Sensitivity analyses were performed using leave-one-out analysis.

We investigated the influence of variables on estimated effects, including depression severity, proportion of untreated patients, type of tracer used, outcome measure, BMI, sex ratio, mean age, and age differences between groups through the use of scatter plots and exploratory mixed-effects models.

## Results

The systematic literature search yielded 11 eligible studies, three of which reported data from other studies (Setiawan et al., 2015; Li et al., 2018b; Turkheimer et al., 2021), resulting in final inclusion of eight studies with 238 MDD patients and 164 healthy controls (Table 1).

**TABLE 1:** Key data of selected studies.

Study		Tracer	Outcome measure	Pharmakokinetic model	Healthy controls				MDD Patients						
					
Author(s)	Year				*n*	F	Age	BMI	*n*	F	Age	BMI	HDRS	% untreated	Medication
Hannestad et al. (2013)	2013	[^11^C]PBR28	V_T_	MA1	10	6	39	26.7	10	5	37	25.4	20*	**	2 patients on stable medication dose or suspended medication (>4 weeks)
Su et al. (2016)	2016	[^11^C]PK11195	BP_ND_	SRTM (SVCA)	13	8	68	Missing	5	3	73.2	Missing	8*	**	Not specified
Richards et al. (2018)	2018	[^11^C]PBR28	V_T_/*f*_P_	2TCM	20	10	31.6	26.01	28	11	39.2	27.8	24*	42.86	16 patients on stable medication, 12 patients medication free (>2 weeks)
Holmes et al. (2018)	2018	[^11^C]PK11195	BP_ND_	SRTM (cerebellar GM)	13	6	33	23	14	7	30	23	20	100	Medication free > 8 months
Setiawan et al. (2018)	2018	[^18^F]FEPPA	V_T_	2TCM	30	14	33.2	24.9	50	31	34.45	24.6	21.05	38	19 patients medication free (>6 weeks)
Li et al. (2018a)	2018	[^18^F]FEPPA	V_T_	2TCM	30	15	27.4	24.3	50	25	28.7	24.5	20.6	**	Medication free
Schubert et al. (2021a)	2021	[^11^C]PK11195	BP_ND_	SRTM (SVCA)	25	14	37.3	24.2	51	46	36.2	27.2	18.5	17.5	9 patients medication free,
Joo et al. (2021)	2021	[^11^C]PK11195	BP_ND_	SRTM (SVCA)	23	10	24.5	23.6	30	17	24.6	23.6	24.3	100	Medication free/treatment naive
Sum: 8					164				238						

MDD, major depressive disorder; F, number of female participants; HDRS, Hamilton Depression Rating Scale (17-item); BMI, body mass index; V_T_, distribution volume of the tracer; V_T_/f_P_, V_T_ corrected for plasma-free fraction; BP_ND_, binding potential; MA1, multilinear analysis; SRTM, simplified reference tissue modeling; SVCA, supervised cluster analysis; 2TCM, two tissue compartment model; GM, gray matter; *equivalent to reported Montgomery Åsberg Rating scale (Leucht et al., 2018); **not reported.

### Anterior cingulate cortex

**FIGURE 2:**
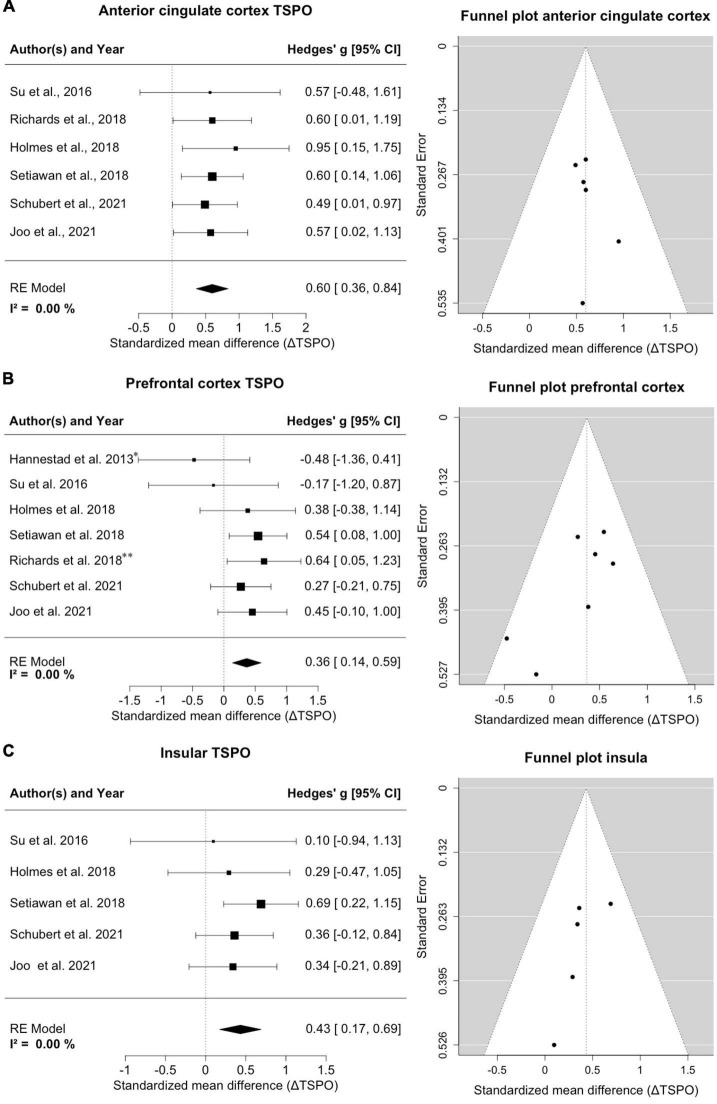
Significant increase of TSPO binding in anterior cingulate cortex (ACC), prefrontal cortex (PFC) and insula in MDD patients. **(A)** A forest plot of TSPO binding in the ACC clearly shows an effect size of 0.60, 95%CI: (0.36, 0.84). The corresponding funnel plot is displayed on the right. **(B)** The forest plot of TSPO in PFC displays an effect size of 0.36, 95%CI: (0.14, 0.59). *Data of frontal cortex; ^**^data of subgenual prefrontal cortex. On the right is the funnel plot of estimates of prefrontal cortex. **(C)** A forest plot for TSPO binding in the insula shows an effect size of 0.43, 95%CI: (0.17, 0.69). Alongside the corresponding funnel plot is displayed.

Six studies with a total of 178 MDD patients and 124 healthy controls reported on TSPO binding in anterior cingulate cortex (Figure 2A). Effects were clearly homogeneous (*I*^2^= 0%), and the results disclosed an exclusion of zero in the confidence intervals [0.60; 95%CI: (0.36, 0.84)] strongly supporting an elevation of TSPO binding in the anterior cingulate cortex in MDD patients. This result was robust to leave-one-out analysis. The funnel plot for anterior cingulate cortex showed symmetry. Exploratory mixed-effects models on investigated variables revealed no significant influences of age, gender, etc. Furthermore, investigation of scatterplots did not indicate any association between explored variables and estimated effects.

### Prefrontal cortex

Seven studies comprising 188 patients and 134 controls reported on TSPO in regions with the frontal cortex, predominantly the prefrontal cortex (Figure 2B). One study reported on the subgenual prefrontal cortex as a subregion and one reported the entire frontal cortex (Hannestad et al., 2013; Richards et al., 2018); both were compiled into a single frontal brain region. Despite this lumping, there was a distinct homogeneity of effects (*I*^2^= 0%), and the confidence intervals excluded zero [0.36; 95%CI: (0.14, 0.59)], indicating significantly increased TSPO binding sites in the prefrontal cortex in patients. The result remained steady in a leave-one-out analysis. The funnel plot for the frontal cortex was slightly asymmetrical, indicating potential publication bias favoring decreases. Exploratory mixed-effects models on tested/recorded variables did not indicate any significant influences. The scatterplot analysis also indicated no relationship between variables and effect sizes on TSPO binding.

### Insula

Five studies covering 150 patients and 104 controls reported on insular cortex TSPO binding (Figure 2C). Homogeneity of effects was evident (*I*^2^= 0%), and confidence intervals again excluded zero [0.43; 95%CI: (0.17, 0.69)], exposing a clearly upregulated TSPO expression in the insula of patients with MDD. Visual inspection of funnel plot indicated missing studies on the right lower side. The result was robust to leave-one-out analysis and the exploratory mixed-effects models carried out to search for possible confounding variables did not reveal any significant interactions. No relationships were observed in the visual inspection of scatterplots.

### Hippocampus

**FIGURE 3:**
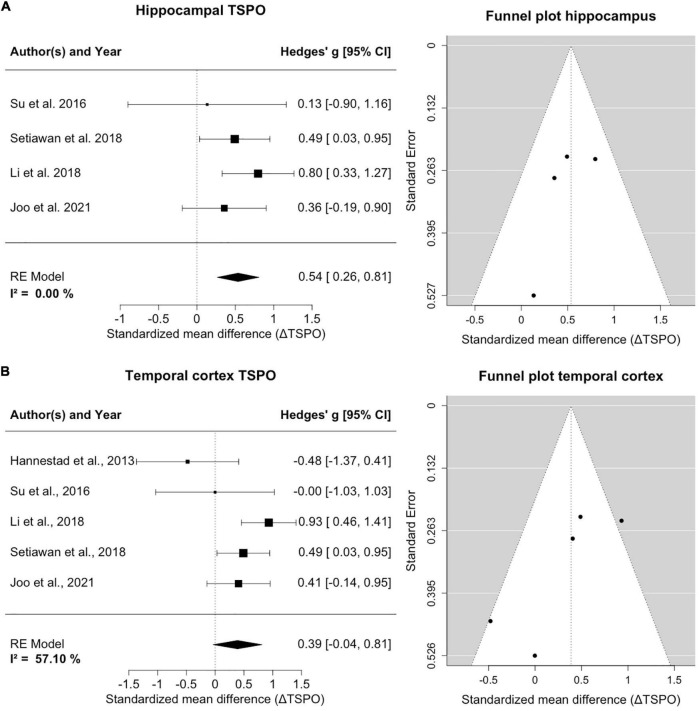
TSPO binding is increased in hippocampus and temporal cortex of MDD patients compared to controls. **(A)** The forest plot for TSPO binding in the hippocampus shows an effect size of 0.54, 95%CI: (0.26, 0.81). The funnel plot of hippocampal estimates is shown on the right side. **(B)** The forest plot of TSPO binding in the temporal cortex indicates an effect size of 0.39, 95%CI: (–0.04, 0.95). The corresponding funnel plot is displayed alongside.

Four studies counting 135 patients and 96 healthy controls report on hippocampal TSPO (Figure 3A). A homogeneity of effects was apparent (*I*^2^= 0%), and there was exclusion of zero in the confidence intervals [0.54; 95%CI: (0.26, 0.81)], indicating elevated TSPO binding in the hippocampus of MDD patients. The funnel plot showed a marginal asymmetry favoring negative results. The hippocampal result was robust to leave-one-out analysis and further exploratory analyses of variables showed no significant influence on estimated effects.

### Temporal cortex

Five studies including 145 patients and 106 healthy controls reported on TSPO binding in the temporal cortex (Figure 3B). An increase of temporal cortex TSPO binding was found, but confidence intervals included zero [0.39; 95%CI: (–0.04, 0.81)]. There was moderate heterogeneity of effect estimates in temporal cortex (*I*^2^= 57%). Leave-one-out analysis revealed that excluding data of Hannestad et al. (2013) resulted in a significant decrease of heterogeneity and a shift of confidence intervals excluding zero [0.57; 95%CI: (0.25, 0.88), *I*^2^= 21%]. We note that, relative to controls, the MDD group of Hannestad et al. (2013) study had the lowest mean BMI, and the largest mean BMI difference between groups, and that the study excluded patients with elevated peripheral inflammatory markers, which may together have biased the results. Visual inspection of the funnel plot suggested a negative publication bias and revealed that smaller studies reported lower effect sizes in the temporal cortex.

## Discussion

The results of this meta-analysis strongly indicate elevated binding of TSPO in all investigated regions in MDD patients vs. healthy controls, which suggests neuroinflammation extending across a range of cortical brain regions previously implicated in executive function (prefrontal cortex), mood (anterior cingulate, hippocampus), sensory processing and homeostatic state (insula), and affective processing (temporal lobe). We note a considerable overlap with brain regions showing reduced perfusion in a meta-analysis of spin-labeling fMRI studies in MDD (Wang and Yang, 2022), and partial concurrence with regions of decreased metabolism to FDG-PET (Su et al., 2014). The present meta-analysis indicates an approximately 29% elevation in the expression of TSPO in the anterior cingulate cortex in a group of more than one hundred MDD patients. The anterior cingulate cortex exhibited the most robust results among investigated regions, with a medium to large effect size, and with five of six studies reporting increased TSPO binding. That *in vivo* TSPO PET finding concurs with *post-mortem* data showing regional microglial activation in the dorsal anterior cingulate cortex of suicide victims with MDD (Torres-Platas et al., 2014; Snijders et al., 2021). The anterior cingulate cortex is considered to be a key structure in circuit models of mood processing and in mediating attention and executive function, and its disruption is thought to impair the ability to process affective information or emotions (Price and Drevets, 2010; Rolls, 2019), which are deficits commonly reported in MDD patients (Naranjo et al., 2011). Furthermore, Li et al. (2018a) identified an association between attentional deficits and increased TSPO expression in the frontal cortex, potentially underpinning the regionally pronounced increase of TSPO availability in patients.

As a key part of the limbic system, the hippocampus is among the brain regions most often studied in mood disorders (Belleau et al., 2019). Our finding of higher TSPO binding in the hippocampus of MDD patients may have some bearing on reports of impaired hippocampal neurogenesis and reduced volume as an anatomic correlate of depressive behavior and cognitive changes (Cole et al., 2011; Chesnokova et al., 2016; Roddy et al., 2019). Similarly, we saw pronounced increases in TSPO-PET signals in the prefrontal cortex, which is also known as a region showing functional and structural changes in depressions (Pizzagalli and Roberts, 2022). Furthermore, a recent review identified the anterior cingulate cortex, prefrontal cortex, temporal cortex, and the insula as the main cerebral regions with disrupted functional connectivity in association with the impaired emotional processing of MDD patients, thus broadly overlapping with regions of increased TSPO PET signal (Li et al., 2022b).

We focused on TSPO PET results in the five brain regions most frequently reported in the eight studies included in our meta-analysis, with a consistent finding of increased TSPO expression in key cortical regions associated with the pathophysiology of depression. A small number of the included studies reported modest differences between groups or even increased TSPO availability in healthy individuals compared to patients, which may contribute to the low effect size estimates in the temporal cortex (Hannestad et al., 2013; Su et al., 2016). Whereby Su et al. (2016) scanned patients with very low MADRS scores and Hannestad et al. (2013) reported relatively higher TSPO expression in healthy individuals in all regions, although that finding was non-significant, with very low sample size, and large inter-subject variability. A few published reports including additional regions likewise indicated a trend toward elevated TSPO expression (Li et al., 2018a; Setiawan et al., 2018), which needs however to be confirmed by studies in large and homogeneous patient samples.

A few exploratory studies have investigated possible factors influencing the effect size in TSPO-PET studies. A [^11^C]PBR28 PET study by Tuisku et al. (2019) found an inverse relationship between tracer V_T_ with BMI in healthy subjects. The same report also showed that women exhibited higher V_T_ than men, and that increasing age was a factor predicting higher V_T_ in the frontal and temporal cortices of MDD patients; we did not recapitulate those findings in our meta-analysis of TSPO-PET studies. In our regional analyses, there were no discernible effects of depression score, age, BMI, percentage of untreated patients, sex distribution, choice of tracer, and the choice of TSPO PET outcome measure. Visual inspection of scatterplots indicated a trend toward higher effect sizes in studies of MDD patients with higher depression scores, which was however not supported by mixed-effects model analysis. We concede that the number of studies in our regional analyses was limited, and propose that a more comprehensive meta-analysis using individual subject data would be necessary to provide a reliable assessment of confounding factors on the availability of TSPO (Plavén-Sigray et al., 2018, 2021). A key finding of Setiawan et al. was that duration of untreated MDD significantly correlated with TSPO V_T_ in three of the examined regions (prefrontal cortex, anterior cingulate cortex, and insula), and that disease duration was a predictor for greater TSPO expression in MDD patients (Setiawan et al., 2018). Available data on disease duration, current episode, and duration of non-treatment in included studies were sparse; only two studies reported current episode duration, and three studies included disease duration as potential confounding variables. An additional factor of interest might be disease-related alterations in regional brain volume, which could lead to artifactual findings of altered TSPO availability. However, one study reporting volumetric brain data found no group differences in regional tissue volumes, despite elevated TSPO availability in patients compared to healthy subjects (Holmes et al., 2018). Furthermore, large multi-center trials reported only small effects of MDD on brain volumetric outcomes (Thompson et al., 2020), which is thus unlikely to account for the differences in TSPO binding in MDD patients compared with healthy controls.

The results of this meta-analysis support the clinical implications of findings from a recent systematic review and meta-analysis (Köhler-Forsberg et al., 2019), which reported that adjunctive anti-inflammatory therapy can alleviate certain MDD symptoms. Moreover, the discipline of clinical psychiatry has long been searching for unambiguous objective diagnostic measures in what may well be heterogeneous disorders. Certainly, the elevation of cortical TSPO binding in MDD patients has an effect size comparable to that seen in our previous meta-analysis showing slightly reduced availability of serotonin transporters in MDD (Gryglewski et al., 2014). However, the present effect size is lower than that reported in a [^11^C]harmine PET study showing increased availability of binding sites for monoamine oxidase A (MAO-A), the enzyme metabolizing serotonin (Meyer et al., 2006). At present, no PET molecular biomarker is pathognomonic of depression, but the composite of results with various tracers might eventually serve to identify molecular sub-types of the disorder, and further investigation might test the hypothesis that there is spatial overlap between increased TSPO binding and altered markers of serotonin innervation and metabolism, or other established molecular imaging results.

TSPO may not only serve as a diagnostic marker in combination with other neuroimaging modalities, but it might also exert as a direct target for putative therapeutic effects in stress-related diseases such as MDD (Rupprecht et al., 2022). While the present results may present microglia as a therapeutic target, a treatment trial with minocycline failed to rectify the elevated TSPO-PET binding in a cohort of patients with treatment-resistant depression (Attwells et al., 2021). On the other hand, findings of a recently published randomized clinical trial suggest that the easily available serum CRP levels could serve as a predictive biomarker to screen for MDD patients who might benefit from add-on minocycline to antidepressant treatment (Nettis et al., 2021). Furthermore, another recent study suggests that specific inflammatory biomarkers that are known to be produced by activated microglia and which correlate with TSPO V_T_, might serve for selection of MDD patients most apt to benefit from augmentative anti-inflammatory therapy (Attwells et al., 2020). Similarly, we recently showed complex relationships between individual plasma levels of the cytokine adiponectin with TSPO-PET results in healthy control and MDD patient groups (Joo et al., 2021).

We note some limitations of this meta-analysis. First, our analysis was limited to the regions that were most frequently reported in molecular imaging studies investigating TSPO in MDD. Some of the included studies involved additional brain regions such as the parietal cortex, the occipital cortex, or non-cortical regions such as the thalamus, amygdala, and putamen (Hannestad et al., 2013; Su et al., 2016; Holmes et al., 2018; Richards et al., 2018; Setiawan et al., 2018). Corresponding data on other brain areas, including the motor cortex and the visual cortex are not yet reported in the literature. Due to the use of different radiotracers and quantification methods, TSPO PET studies can be inherently difficult to compare. By calculating standardized mean differences, we attempted to avoid distortions due to the different measurement scales. Furthermore, we used random-effect models to accommodate the anticipated heterogeneity. However, this approach does not completely eliminate bias from variation between study populations (Lin and Aloe, 2021). As is well known, current TSPO PET tracers indicate only the overall level of microglial activation, but do not differentiate between the anti-inflammatory and pro-inflammatory microglial phenotypes. In addition, TSPO ligands are not entirely specific for microglia, but can indicate other neuroinflammatory changes, including activation of astroglia cells and monocyte-derived macrophages (Cosenza-Nashat et al., 2009). However, the preponderance of the TSPO-PET signal in brain tissue is likely indicative of microglial activation (Setiawan et al., 2018). PET imaging studies using newer radioligands for biomarkers of inflammation other than TSPO, such as monoamine oxidase B, cyclooxygenase, colony stimulating factor 1 receptor, and the purinergic P2X_7_ receptor may eventually confirm the state of neuroinflammation in MDD (Narayanaswami et al., 2018; Zhou et al., 2021). We concede that some regional analyses in this meta-analysis involve a small number of studies, which limits their statistical power and may have impeded detection of a publication bias. A further limitation is that most studies included in this meta-analysis used the first-generation radioligand [^11^C]PK11195, which has a relatively low specific binding signal and brain uptake (Kreisl et al., 2010; Cumming et al., 2018; Kobayashi et al., 2018), albeit not having the TSPO allelic dependence of second-generation tracers. In this context a meta-analysis on TSPO-PET in schizophrenia reported that differences between patients and healthy subjects were only apparent from studies using [^11^C]PK11195, without any such difference evident in the studies using second-generation TSPO radioligands like [^11^C]PBR28 and [^18^F]FEPPA, noting that these findings were driven by small studies with low variability in outcomes (Marques et al., 2019). Of interest, a recent study revealed that the Korean population seems to lack the polymorphism of the rs6971 allele, thereby suggesting a lower impact on second-generation TSPO PET tracer binding in this patient group (Lee et al., 2022). Analytic procedures also affect the signal-to-noise ratio of TSPO PET measurement, in the absence of a valid reference region for calculating the binding potential (BP_ND_) (Cumming et al., 2018). In an effort to select the most appropriate reference region, most of the included studies reported BP_ND_ as the outcome measure, as calculated by the simplified reference tissue model using a supervised clustering approach to segment the reference region (Turkheimer et al., 2007; Boellaard et al., 2008; Yaqub et al., 2012; Schubert et al., 2021b). Holmes et al. relied on cerebellar gray matter as a pseudoreference region due to the lower variance in BP_ND_ results compared to the data-driven approach (Kropholler et al., 2007; Holmes et al., 2018). The gold standard endpoint is therefore total distribution volume (V_T_), which calls for serial arterial sampling with correction for tracer metabolism (Wimberley et al., 2021). The most common pharmacokinetic model to calculate V_T_ in the included studies was a two-tissue compartment model, which appears to be the most feasible for second-generation TSPO ligands (Fujita et al., 2008; Rusjan et al., 2011). Ichise et al. (2002) and Hannestad et al. (2013). relied on a multilinear analysis to calculate V_T_ because of lower standard errors in estimates compared to the two-tissue compartment model. Notwithstanding, mixed-effects models did not indicate any effects of radioligand type or outcome measures on the finding of increased TSPO expression in MDD patients.

In summary, this systematic meta-analysis clearly highlights prior findings of increased TSPO binding in MDD patients compared to healthy controls in the broadest sample of TSPO PET data yet assembled. In MDD patients, we saw an ∼18% relative increase in TSPO expression, which was present in all investigated brain regions, and with effect sizes of comparable magnitude to those in previous PET studies of availability of serotonin transporters and monoamine oxidase A binding in MDD (Meyer et al., 2006; Gryglewski et al., 2014). The findings were robust to depression severity, BMI, medication status, and other explored variables, and statistical analysis indicates that the effect was driven by significant increases of TSPO in one third of the MDD patients. Hence, TSPO elevation is indeed a feature of MDD, and future *in-vivo* studies with novel radioligands targeting neuroinflammation may prove to support the occurrence of a central inflammatory component in MDD.

## Data availability statement

Processed data is available from the authors upon reasonable request. Please contact the corresponding author for any questions or requests.

## Author contributions

BE and GG performed data collection and analysis. PC and J-HK proposed the study. All authors contributed to the interpretation of results and drafting of the manuscript and approved the final version of the manuscript.
